# Clinical Assessment of SARS-CoV-2 Antibodies in Oral Fluids Following Infection and Vaccination

**DOI:** 10.1093/clinchem/hvad169

**Published:** 2023-12-01

**Authors:** Christopher D Heaney, Heidi Hempel, Kate L DeRosa, Ligia A Pinto, Nicholas J Mantis

**Affiliations:** Department of Environmental Health and Engineering, Johns Hopkins Bloomberg School of Public Health, Baltimore, MD, United States; Department of Epidemiology, Johns Hopkins Bloomberg School of Public Health, Baltimore, MD, United States; Department of International Health, Johns Hopkins Bloomberg School of Public Health, Baltimore, MD, United States; Vaccine, Immunity and Cancer Directorate, Frederick National Laboratory for Cancer Research, Frederick, MD, United States; Division of Infectious Diseases, NewYork State Department of Health, Wadsworth Center, Albany, NY, United States; Vaccine, Immunity and Cancer Directorate, Frederick National Laboratory for Cancer Research, Frederick, MD, United States; Division of Infectious Diseases, NewYork State Department of Health, Wadsworth Center, Albany, NY, United States

## Abstract

**Background:**

SARS-CoV-2 variants continue to circulate globally, even within highly vaccinated populations. The first-generation SARS-CoV-2 vaccines elicit neutralizing immunoglobin G (IgG) antibodies that prevent severe COVID-19 but induce only weak antibody responses in mucosal tissues. There is increasing recognition that secretory immunoglobin A (SIgA) antibodies in the upper respiratory tract and oral cavity are critical in interrupting virus shedding, transmission, and progression of disease. To fully understand the immune-related factors that influence SARS-CoV-2 dynamics at the population level, it will be necessary to monitor virus-specific IgG and SIgA in systemic and mucosal compartments.

**Content:**

Oral fluids and saliva, with appropriate standardized collection methods, constitute a readily accessible biospecimen type from which both systemic and mucosal antibodies can be measured. Serum-derived IgG and immunoglobin A (IgA) are found in gingival crevicular fluids and saliva as the result of transudation, while SIgA, which is produced in response to mucosal infection and vaccination, is actively transported across salivary gland epithelia and present in saliva and passive drool. In this mini-review, we summarize the need for the implementation of standards, highly qualified reagents, and best practices to ensure that clinical science is both rigorous and comparable across laboratories and institutions. We discuss the need for a better understanding of sample stability, collection methods, and other factors that affect measurement outcomes and interlaboratory variability.

**Summary:**

The establishment of best practices and clinical laboratory standards for the assessment of SARS-CoV-2 serum and mucosal antibodies in oral fluids is integral to understanding immune-related factors that influence COVID-19 transmission and persistence within populations.

COVID-19 continues to persist globally due to the extremely high transmissibility rates of SARS-CoV-2 and its ever-evolving variants of concern. Severe COVID-19 disease is principally associated with infection of the lower lung and was responsible for the devastating outcomes of infection in the earliest days of the pandemic. The first-generation mRNA and subunit SARS-CoV-2 vaccines elicited high levels of spike-specific immunoglobin G (IgG) antibodies in circulation that dramatically reduced the risk of severe COVID-19. However, the durability of such responses is considered suboptimal, thereby necessitating routine boosters. Moreover, the emergence of SARS-CoV-2 variants, including the highly evasive Omicron, has altered the immunological landscape and necessitated the deployment of second-generation bivalent (original antigen plus Omicron BA.4/BA.5) and third-generation monovalent (Omicron XBB.1.50) COVID-19 vaccines. Despite these efforts, SARS-CoV-2 remains a life-threatening infection in many populations such as the elderly, cancer patients, pregnant women, and the immune-compromised (1–3).

There is increasing recognition that the mucosal immune system and local secretory antibodies in oral fluids (OF) and nasopharyngeal (NP) fluids may be a barrier to reinfection as well as an impediment to SARS-CoV-2 shedding and transmission, especially in the case of Omicron (4, 5). Following inhalation, SARS-CoV-2 replication can occur in the cells that line the upper respiratory tract, including the salivary glands that express the requisite SARS-CoV-2 receptors ACE2 and TMPRSS (6). Release of the virus into oral and NP fluids occurs in symptomatic and asymptomatic individuals alike and can contribute to person-to-person transmission (7, 8). From an immunological standpoint, it is postulated that only local antibodies in OF and secretions of the upper respiratory tract can engage with incoming SARS-CoV-2 virions and impede outgoing virus destined for person-to-person transmission. Therefore, assessing compartment-specific SARS-CoV-2 antibody levels in OF and NP may be the key to understanding virus transmission.

OF represent an easily accessible biospecimen type to interrogate both systemic and mucosal immune responses (9). Gingival crevicular fluids (GCF) and saliva each contain serum-derived IgG and immunoglobin A (IgA) as the result of transudation through the epithelial barrier. Secretory immunoglobin A (SIgA), which is produced locally following mucosal infections, is present almost exclusively in saliva and passive drool. However, quantitative analysis of serum- and mucosa-derived, antigen-specific antibodies in OF is intrinsically and extrinsically challenging (9). In this mini-review, we argue for the need for the adoption of standards, highly qualified reagents, and best practices to ensure that clinical science is both rigorous and comparable across laboratories and institutions. We discuss the need for better understanding of how sample stability, collection methods, and other factors that affect measurement outcomes can impact clinical applications of OF to improve SARS-CoV-2 outcomes and interventions within populations at large. Because bulk collection of liquids from the oral cavity using commercially available collection devices include a mixture of GCFs and saliva, we use the term OF throughout this article.

## Assessing Immunity to SARS-CoV-2 in OF

**Fig. 1.**
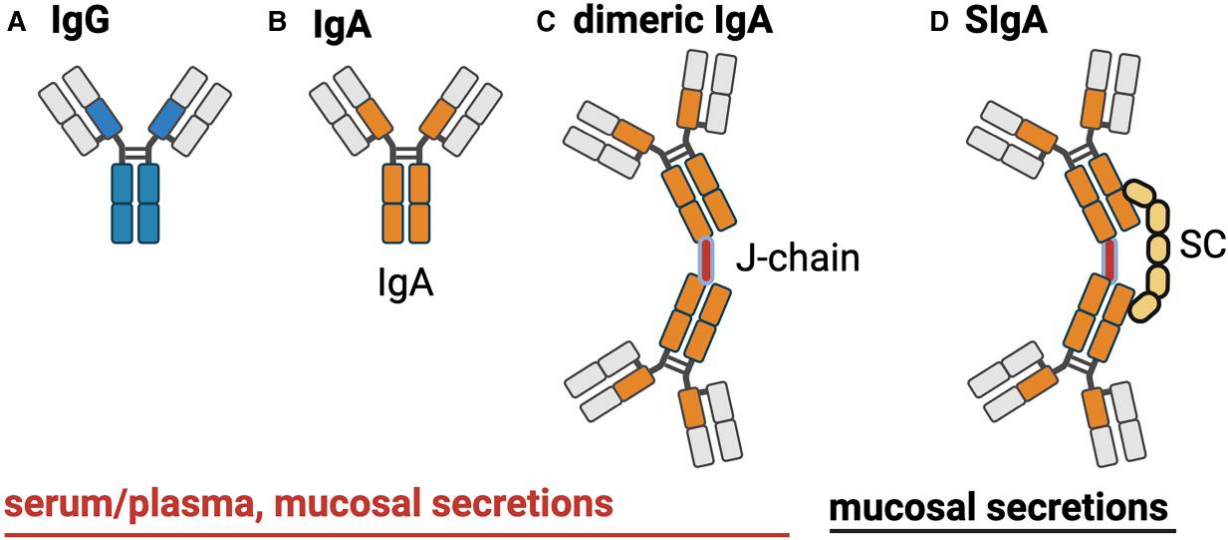
Immunoglobulin types and forms found in OF. Serum derived (A) IgG and (B) monomeric IgA are found in saliva and OF because of transudation. Mucosa-derived (C) dimeric IgA consists of 2 IgA monomers joined together by J chain. Dimeric IgA is actively transported across certain epithelia in the oral cavity by the polymeric immunoglobulin receptor and delivered into OF as (D) SIgA. Secretory component is a proteolytic cleavage product of polymeric immunoglobulin receptor and remains bound to dimeric IgA. For immunoassays, dimeric IgA and SIgA can be differentiated from serum-derived IgA using anti-J chain or anti-SC specific antibodies. Created with BioRender.com.

OF contain a mixture of IgG, IgA, and, to a lesser extent, immunoglobin M at concentrations ranging from <0.1 to >100 mcg/mL (9). IgG antibodies are primarily derived from serum and accumulate as the result of transudation across periodontal epithelium and other oral mucosal tissues (Fig. 1). IgA, on the other hand, can arise locally from resident mucosa plasma cells that secrete dimeric and polymeric forms of IgA into the interstitium. Dimeric IgA (dIgA) and polymeric IgA, but not serum-derived monomeric IgA or IgG, are transported by the polymeric immunoglobulin receptor across salivary gland epithelia and released into saliva and OF. A fragment of the polymeric immunoglobulin receptor, known as secretory component (SC), remains covalently associated with IgA after transport. The complex between dIgA and SC is referred to as SIgA (Fig. 1). SC imparts important biological properties to IgA that are not shared with IgG, most notably enhanced conformational stability, protease resistance, and increased half-life in mucosal secretions including the oral cavity (10, 11). SIgA antibodies constitute not only a hallmark of locally produced antibodies but also a formidable barrier against virus infection.

Determining the extent to which different types of antibodies (e.g., IgG, SIgA) in OF contribute to preventing SARS-CoV-2 infection has implications for clinicians and researchers alike. There is emerging evidence that preexisting antibodies (a result of vaccination or prior infection) in OF and NP lavage play a decisive role in limiting SARS-CoV-2 reinfection with emerging variants of concern like Omicron (12–15). In theory, the same population of antibodies may also coat infectious virions and, when packaged in aerosols, limit their potential transmissibility (16).

From a clinical standpoint, threshold values of spike- and nucleocapsid-specific IgG and/or SIgA in mucosal sites may serve as more specific and accessible indicators of immunity compared to immunoglobulins found in serum/plasma, potentially influencing decisions about the need for booster vaccinations in high-risk and vulnerable populations (17, 18). From the vantage point of vaccine research, an outsized role for SIgA in immunity to SARS-CoV-2 would justify investments in intranasal vaccine platforms designed to target mucosal-associated lymphoid tissues and stimulate local humoral (and cellular) responses (4, 19). However, claims about the importance of serum- and local-derived antibodies in immunity to SARS-CoV-2 in human populations can only be substantiated if methods and reagents associated with the collection and analysis of OF are optimized, standardized, and harmonized across laboratories. At present, no such standards exist for SARS-CoV-2.

## Lessons from Oral Immune Surveillance Studies of Human Papilloma Virus

There is a long-standing interest in studying mucosal secretions as a non-invasive alternative to serology in assessing immunity to a range of infectious agents, including the human papilloma virus (HPV) (20). Veinous blood draws are more invasive than OF or NP collections and do not measure local antibody levels at the primary site of infection (the genital and oral mucosa) (21). Parker et al. compared antigen-specific IgG in OF to serum in males and females that had been immunized with a licensed HPV vaccine, Gardasil (22). The ELISA assay employed had been optimized for detection of oral HPV antibodies in mouthwash rinses and saliva samples (21). Although normalized mucosal IgG levels correlated well with serum levels, concentrations of HPV-specific IgG levels in OF were much lower than those in serum, suggesting antigen-specific IgG declines faster in OF compared to serum (23). Simultaneously, the number of individuals with detectable levels of mucosal IgG also decreased significantly faster than serum IgG, possibly suggesting a significant loss of detectability of antibodies to HPV in saliva (22). As saliva has 10- to 100-fold lower levels of IgG compared to the blood (largely due to the fact that detected IgG in saliva is transudated from blood), this loss of detectability potentially increases variability in IgG measurements in the saliva compared to blood and also warrants methods development to improve the lower limit of detection and sensitivity of oral fluid immunoassays (22, 24). Indeed, we and others have noted that levels of antibodies can be highly variable between antigen-specific IgG in blood and saliva (21, 22). Sources of variability include method or site of collection, whether saliva production is stimulated or unstimulated, whether the site is washed prior to collection, differences in assays and protocols, and patient behavior before or during collection (21, 24, 25). Such variables may be further complicated by possible oral inflammatory sequelae associated with COVID-19 that may result in increased serum transudation into OF (26).

### Applications of Oral Fluid To Understanding Immunity to SARs-CoV-2

The technical challenges associated with the use of OF must be confronted as the research and clinical communities explore this biospecimen as a means of evaluating mucosal immunity to SARS-CoV-2 (4). Studies to date aimed at understanding SARS-CoV-2 mucosal immunology have encountered the same issues as those that arose in HPV. For example, some studies have shown that IgA is detectable in OF of patients currently or previously infected with SARS-CoV-2, but the levels are highly variable and decline rapidly (15, 27, 28). It has also been shown that, while anti-Spike IgG is present and relatively stable over 9 months in OF of convalescent patients (100% at 3 months to 87.5% at 9 months post-symptom onset), only 55% of convalescent patients were positive for salivary anti-Spike IgA at 3 months post-symptom onset, with levels gradually declining to 9.7% by 9 months (29).

Such discrepancies are related to both extrinsic and intrinsic variables associated with working with OFs such as saliva and GCF. Extrinsic factors include the various methods of collection, testing platforms, and isotype detection reagents. For example, most investigators infer levels of secretory antibodies (e.g., SIgA) in OF from the use of IgA-specific secondary antibodies, rather than detection of hallmarks of mucosal IgA, namely anti-J chain and anti-SC (30). Granted there is a strong correlation between IgA and SIgA in OF, but failure to fully account for the different forms of IgA confounds claims about the roles of mucosa-derived antibodies elicited following infection and vaccination (9, 12).

Intrinsic factors affecting salivary IgG and IgA levels can include patient age, oral health, and disease severity. One study found that IgG and IgA levels in saliva were significantly lower in children than in adults, and infection-stimulated salivary IgG seroconversion rates were higher in adults than in children (with no significant difference in salivary IgA seroconversion rates) (31). Meanwhile, SARS-CoV-2 neutralizing activity in the nasal cavity is associated with both lower disease severity and higher nasal IgA response (31, 32). These results may indicate that IgA levels increase in response to disease severity rather than the opposite. Alternatively, timing of an increase in mucosal IgA may determine whether there is protection vs disease progression and further activation of mucosal IgA.

The relationship between salivary antibody levels and COVID-19 vaccination are even more complex, with the route of exposure being intramuscular rather than mucosal and IgA levels presenting at much lower levels (7, 33). Many assays (including Luminex-based assays) report low sensitivity and/or specificity for IgA in saliva, meaning that the low levels induced by vaccination may result in significant loss of signal, increasing measurement variability and background (33, 34). In addition, different vaccination platforms also seem to result in significantly different mucosal immunoglobulin profiles. Nahass et al. found that individuals vaccinated with the adenovirus-based Ad26.COV2.S had very low levels of antibodies in their saliva, while immunoglobulins levels in the saliva of mRNA-based vaccines were robust (33). In addition, Ad26.COV2.S recipient OF showed little to no neutralizing activity, while mRNA-vaccine recipient saliva successfully neutralized the virus. In fact, participants receiving 2 doses of mRNA vaccine were the most likely to have neutralization levels comparable to those seen in convalescents (33). However, neutralizing activity was both highly transient and lower in the saliva as compared to the blood. Interestingly, another adenovirus-based vaccine, ChAdOx1/nCOV-19, did induce salivary-neutralizing levels similar to the mRNA vaccines (33).

The kinetics of IgA production in the saliva may also be starkly different than salivary IgG. Sheikh-Mohamed et al. reported that IgG in the saliva was significantly boosted by a second dose of mRNA vaccine followed by a steady decline over 3 to 4 months (similar to in serum), while the SIgA response significantly and rapidly decreased by 2 weeks post-second dose (15). In this study, only 30% of vaccinated participants remained detectably positive for SIgA by this time-point and at levels significantly lower than in individuals who had been infected. Interestingly, however, those levels in the 30% appeared steady thereafter (15). It is worth noting that this difference in responses may be because salivary IgG is mainly transudated from the serum rather than mucosally derived. Therefore, there is a possibility that serum Ig kinetics are different in response to boosting than in the mucosa, rather than the difference lying in IgG vs IgA production. This difference might be parsed out via normalization against blood proteins such as hema, transferrin, or albumin, or even against total IgG (24, 35). These normalization steps will also ensure that the levels being studied are due to mucosal production, rather than blood contamination due to gingival disease, changes in salivary secretion, and so on.

## Collection of OF—Challenges and Opportunities

The collection of OF can be completed unsupervised at home and returned to clinics via the mail, thereby eliminating the need for health care staff or medical facilities (36, 37). Self-collection using an Oracol device has also improved large-scale population-level surveillance of infectious diseases due to pain-free collection methods and elimination of the need to travel to a medical office. Given that populations in rural locations or of lower socioeconomic status may not have access to medical facilities, self-collection may provide a more representative sample of the general population (37).

Despite these benefits, the use of OF for immunological surveillance poses challenges (38). Different collection methods harvest fluids from different glands, resulting in different proportions of mucosal secretions and transudate. Brushing the gum line with a swab device yields OF principally comprised of GCF, which is enriched with IgG from serum transudate but also captures mucosally secreted IgA (24, 27). Other collection methods, such as passive drool, allow for more uniform collection of whole saliva but result in highly diluted immunoglobulin levels that can impact the sensitivity of testing (38). As a result, oral fluid-based antibody profiling tends to be considered less accurate and have lower sensitivity than serum-based serological methods (27). In addition, precollection variables can be introduced through autoimmune disease, drinking, smoking, rinsing, gum bleeding due to oral disease, bacterial load, or taking oral medications (25). Consequently, antibody levels in oral fluid are not only highly variable from person to person but may also vary within a given individual throughout the course of a day. In summary, many different elements appear to contribute to and influence salivary immunoglobin levels. Although controlling for all variables may prove extremely difficult, building questionnaires that collect information regarding all these health and clinical variables will be an integral part of any mucosal humoral immunity study.

The logistics of saliva transportation from the patient to the laboratory (as well as storage) also constitutes a challenge, as little has been reported in terms of sample stability in transit (27, 37, 39, 40). One report suggests that salivary proteins and macromolecules degrade at room temperature within 30 minutes of sample collection (41). There is a consensus that best practices will require immediate processing and aliquoting to avoid loss and minimize impact of freeze-thaws. A study mimicking the conditions of oral fluid (collected using an Oracol swab) shipment from the UK Communicable Disease Surveillance Centre found total IgG and rubella-specific IgG to be stable at both 10°C and 20°C for up to 10 days (42). In the case of COVID-19, passive drool from a single patient with detectable SARS-CoV-2 IgG antibodies was stored at 4°C and 27°C for 6 days without the addition of stabilizers to mimic time spend in transit when mailed. Results showed no significant change in the level of salivary antibodies at either temperature (39). While this study suggests oral fluid samples are a stable specimen source for IgG, further research with a strong study design is needed to fully understand the impact of sample storage conditions on salivary immunoglobulins for seroprevalence studies and other biomarkers of interest. There is an urgent need to establish standardized collection (with or without preservatives, such as protease inhibitors), transportation, processing, and storage standard operating procedures, along with standardized questionnaires and standardized data vocabulary.

## Standardization of Methodologies

A diverse range of oral fluid collection approaches and devices have been employed during the COVID-19 pandemic to estimate SARS-CoV-2 specific antibody responses, potentially adding to the variation in data and conclusions. Passive drool has been a popular method due to its early acceptability, use, and integrity to support broad population-based SARS-CoV-2 reverse transcriptase real-time polymerase chain reaction testing as part of routine asymptomatic COVID-19 screening and surveillance programs (43). Passive drool or whole unstimulated saliva involves participants following instructions to expel saliva passively as drool from their oral cavity into any number of sterile collection tubes (e.g., SalivaBio) (Table 1). Collection of passive drool may be unfeasible in populations who cannot readily follow verbal instructions to spit on command (e.g., newborns and infants) or those who are nonresponsive (e.g., the elderly, individuals who are sedated, intubated, or lethargic). To address these challenges with oral fluid collection in such special populations, alternative methods can be employed that use a swab or sponge to harvest antibody-enriched oral mucosal transudate and/or GCF. Examples of swab- or sponge-based approaches include simple sponge-on-a-stick designs with no added buffers, preservatives, stabilizers, or reagents (e.g., Oracol + S14) and absorbent foam pads that may be impregnated with reagents and placed into a storage buffer to enhance the recovery and/or stability of antibodies (e.g., OraSure Oral Specimen Collection Device) or produce a visual sign that oral fluid of a sufficient volume and quality has been collected (e.g., Versi-SAL, Oasis). Other collection devices lack a stick or collection handle and involve insertion of an absorbent wicking cylindrical pad or sponge into the oral compartment, with specific placement instructions that can be tailored to target recovery of fluid from specific regions of the oral cavity [e.g., submandibular, sublingual, or parotid fluid (e.g., Salivette, Sarstedt)]. Table 1 summarizes exemplary collection devices and approaches that have been employed for oral fluid collection with SARS-CoV-2 antibody response measurements.

**Table 1.** Overview of commercial oral fluid collection devices.

Specimen type	Swab/spoon	Ab conc.^a^	Stablizer	Special pop.	Manufacturer/vendor
Passive drool					
SalivaBio	No	+	No	None	Salimetrics LLC
SpeciMAX	No	+	No	Not rec. <6 y	Thermo Fisher
SalivaDirect	No	+	No	Not rec. <6 y	Yale University
OMT/GCF					
OraSure	Yes	++	Yes	None	OraSure Technologies
Oracol + S14	Yes	+++	No	None	Malvern Medical Developments
Oasis Versi-SAL	Yes	++	No	None	Oasis Diagnostics
SM, SL, parotid					
Salivette	Yes	++	No	None	Sarstedt
SalivaBio SOS	Yes	++	No	Adult	Salimetrics LLC
SalivaBio SCS	Yes	++	No	Child	Salimetrics LLC
SalivaBio SIC	Yes	++	No	Infant	Salimetrics LLC

Abbreviations: Ab, antibody; OMT, oral mucosal transudate; SM, submandibular; SL, sublinguial. **^a^**Ab conc. +++, most concentrated to +, least concentrated.

The quality of sample resulting from each oral fluid collection approach can be affected by numerous factors. Passive drool can result in larger sample volumes (1 mL or greater); however, the higher volume tends to serve as a dilution factor of antibody signals of interest in oral fluid. Swab-based collection devices are designed to harvest antibody-enriched oral mucosal transudate and/or GCF, which can result in a high concentration of antibody even if the recovered oral fluid sample volume is lower (on average it can be less than 1 mL). The timing of consumption of foods, drinks, and/or medications prior to specimen collection, time of day, age of the individual, and hydration status can all affect the total oral fluid volume recovered as well as the concentration of antibody and other substances that can interfere with antibody measurements.

## Sample Standards and Data Normalization

Other obstacles to accurate and reliable analysis of mucosal immunity are due to the lack of standardization in the field. There is currently no international SIgA standard with which to harmonize data, and there is high interlaboratory variability in methodology, assay reagents, and analytes used to study SARS-CoV-2 mucosal immunity (Table 1). In the case of SARS-CoV-2, antigen-specific SIgA in the mucosa may directly mediate infection prevention. Therefore, saliva and/or nasal sampling would be a direct measure of immunity rather than a proxy for blood-based immunity. This makes these variables even more of a problem, as mucosal measurements would be essential for the determination of the effectiveness of mucosal vaccines (13, 15, 44). Production of secondary standards using pooled saliva samples may be an opportunity to calibrate within studies, until a World Health Organization International Standard becomes available.

## Conclusions

As the focus of SARS-CoV-2 infection and immunity research shifts from severity and hospitalization mitigation to infection prevention, the need to reliably measure immunity in the mucosa is increasing. Previous experience in HPV research, as well as early COVID-19 research, highlights the significant gaps in this field as it impacts clinical chemistry and other clinical laboratories. In this review, we highlighted the key variables that influence sample testing and results, including collection procedures, patient characteristics, disease severity, and laboratory testing differences. We argue for the establishment of best practices guidelines for sample collection and processing, as well as reliable international SIgA standards for the harmonization, comparability, and normalization of data. Without such information the clinical utility of such information will be severely constrained.

## Nonstandard Abbreviations

IgG, immunoglobin G; OF, oral fluids; NP, nasopharyngeal; IgA, immunoglobin A; SIgA, secretory immunoglobin A; SC, secretory component; GCF, gingival crevicular fluid; HPV, human papilloma virus.
